# The value of forensic DNA leads in preventing crime and eliminating the innocent

**DOI:** 10.1016/j.fsisyn.2021.100201

**Published:** 2021-09-23

**Authors:** Ray A. Wickenheiser

**Affiliations:** New York State Police Crime Laboratory System, 1200 Washington Avenue, Building 30, Albany, NY, 12226-3000, USA

**Keywords:** Forensic science, Forensic DNA, Crime lab, Backlog, Awaiting analysis backlog, Cost-benefit, Identification, Comparison, Elimination, Prevention, Investigative leads, Preventable crime, Sexual assault kits

## Abstract

The value of an investigative lead corresponds directly to the increase of the speed at which that lead is provided. A cost-benefit model using sexual assault cases demonstrates the preventative savings of quicker forensic DNA analytical response times by calculating the cost of additional crime committed while cases sit awaiting analysis to commence. Calculations are provided per analyst day and with estimated U.S. nationwide impacts. With the elimination of the awaiting analysis backlog, crimes could be prevented, as well as justice better served to those wrongfully suspected. A case study demonstrates the value of timely forensic DNA analysis for sexual assault cases. A wrongfully accused individual identified by eyewitness testimony was eliminated by forensic analysis, while a very similar appearing recidivist perpetrator was included in a subsequent DNA comparison.

## 1 Background

Forensic laboratories provide three broad categories of analyses: identification, comparison, and investigative leads. Each analysis type has attributes that provide value to solving particular types of crimes, thereby adding value by reducing the risk posed to society by providing objective data to guide decisions. In many instances, the suspect in a case is known. Providing timely forensic analysis assists in substantiating or negating criminal charges, frequently enables plea bargains and more efficient and effective administration of justice. Where suspects are not known, value is added by assisting the development of persons of interest for further investigation. In either instance, defining the value of the contribution of forensic analysis assists in determining the appropriate target response time, and hence the required allocation of resources necessary to achieve that goal.

Toxicology and Drug Chemistry sections are commonly associated with the first category of forensic analysis, which is identification. Typically, a suspect is arrested on suspicion of driving under the influence of a drug or having a potential controlled dangerous substance in their possession. In other cases, an individual is deceased, and analyses are relied upon to determine the manner of death for drug overdoses. In each of these instances typically a known individual is the suspect or subject. As charges may be pending the outcome of the forensic analysis to confirm the presence and/or quantity of a substance in question, court proceedings are frequently delayed. Similarly, a coroner’s certificate often awaits confirmation of the presence of a particular substance. In these types of cases there is a reasonable suspicion of a crime, hence these crimes are recognized as requiring forensic analysis. This includes impairment, presence of a suspicious looking white powder, the presence of drug paraphernalia and other corroborating evidence. The cost of delayed forensic results due to a backlog of cases awaiting analysis to commence lies in delayed justice and inefficiency of the judicial system. Suspects incorrectly charged can have grievous harm associated with the delayed or ineffective machinations of the justice system, including potentially losing child custody, loss of employment, home foreclosure and inappropriate incarceration [1,2]. These are in addition to the stress, shame, denigration and loss of dignity and social standing associated with being charged of a criminal offense.

Firearms and Latent Print sections frequently provide analyses in the comparison category of analysis. Some number of identification analyses are also provided in the Firearms Section, such as determination of prohibited firearms categorization, or assessment of caliber, make and model of a firearm that may have fired a particular projectile or expended cartridge as an investigative lead. However, a large percentage of Firearms Section analyses compare items found at crime scenes, such as bullets and cartridge casings to a set of known exemplars obtained from firearms in question. Prior to the advent of large databases and algorithms that permit rapid and accurate searches, analysts were limited to directly comparing items from a crime scene (questioned) to a suspect (known). In the case of the Latent Print Section, fingerprints at a crime scene are located, developed, and compared to known suspect exemplars, providing value to investigators both for inclusionary and exclusionary information. This information is of particular value if the latent print was located in an incriminating position, for example at the point of entry to a crime scene or upon a weapon handle.

Comparison analyses graduate into the third type of analyses, investigative leads, when the suspect is not known to investigators, or initial suspects have been eliminated. The application of forensic databases provides a large body of known exemplars as a basis for comparison, the difference being that the lead provided is frequently one that may have been previously unknown to investigators. Commonly used forensic databases include the National Integrated Ballistics Information Network (NIBIN), the Automated Fingerprints Identification System (AFIS) and the National DNA Index System (NDIS). This third category of investigative leads provides an enhanced value to investigators, as resources were often spent investigating incorrect suspects prior to the new person of interest now linked with objective science-based data. At this point, with a forensic laboratory report in hand, investigators seek to confirm or refute new hypotheses, as case circumstances such as location, alibi and other types of evidence are weighed in light of new information.

The NDIS system was created in 1994 with the passage of the DNA identification act [3]. NDIS is comprised of a number of indices, which include a database of offenders and arrested individuals. A DNA profile developed from biological material deposited by the perpetrator of a crime, known as forensic DNA, can be used to searched against profiles of these known individuals to develop investigative leads. Forensic profiles can also be searched among each other, to determine trends of crimes committed by the same individual. Prior to the creation of NDIS, a suspect was required to develop a profile for direct comparison. As of June 2021, the NDIS contained over 14,626,360 offender profiles, 4,401,111 arrestee profiles, 1,117,593 forensic profiles and produced over 570,786 hits assisting more than 557,734 investigations [4]. The success of NDIS demonstrates the ability of databases of known suspects to provide leads where associations were previously unknown. Hence, without NDIS, many crimes have been detected and prevented, interrupting recidivist offenders and preventing additional offenses.

While forensic evidence has very positive features of data and objectivity to back findings, it seldom stands alone without other corroborative evidence. The circumstances surrounding the crime must fit the new forensic evidence. A person of interest may have a very valid reason for being associated with a particular item, individual or location by a latent fingerprint inclusion, such as a worker who had recently completed repairs at a crime scene location prior to the crime being committed. A thorough investigation will include this necessary detailed follow up, demonstrating the true perpetrator had opportunity, access, and motive. Associative evidence such as hairs, fibers, latent prints, and many biological materials cannot be dated as to exactly when they were deposited, therefore alibis and corroborating evidence are valuable pieces to completing the crime puzzle. While physical evidence can associate an individual with another person or a crime scene, it can seldom prove a case without supporting evidence.

## 2 Business case

As investigations are frequently awaiting forensic analytical results to proceed, or worse, are expending resources on the incorrect line of investigation, it is critical to provide prompt analytical results for accurate investigative direction. While the cost of delayed justice and justice system inefficiencies are significant, it is challenging to calculate direct costs. Hence, focus on no suspect sexual assaults has served as a surrogate model to demonstrate overall forensic value. No suspect sexual assaults are used as a model as by definition the survivor of the crime cannot provide a description of the assailant. Frequently masks may be used, or drugs used to facilitate the crime, hiding the suspect’s identity. Sexual assault cases frequently have significant biological materials deposited that are probative to the nature of the crime, the suspect’s semen. Semen is a robust biological fluid which is frequently deposited in significant quantity; hence no suspect sexual assault cases serve as an excellent model to determine cost benefit.

Estimates have been provided to include both a conservative and liberal range of the cost of crime to the individual and society, as a range of cost estimates have been calculated for no suspect sexual assault cases [[5], [6], [7], [8], [9], [10], [11], [12]]. Not included in these estimates is the preventable cost of the unapprehended offender permitted to continue to commit additional crimes while a solvable case sits awaiting analysis to start. Using this additional information of time sensitivity and opportunity cost, a new model has been built that will expand upon previous versions to include the cost of a delayed forensic response. Using the model of no-suspect sexual assaults will permit the calculation of the cost of an awaiting analysis backlog.

Each day that goes by without an identifying investigative lead represents additional time for a recidivist perpetrator to commit the same or escalating crimes upon new victims. Providing forensic DNA analysis immediately upon submission of a case to a forensic laboratory enables a lead to be provided to investigators as quickly as technology permits. The time a case sits awaiting analysis to commence is time an assailant remains at large, perpetrating the same crime upon new victims. A perpetrator is said to commit 26.22 sexual offenses over a 3.69-year career with 7.1 assaults per year, which could be prevented by forensic analysis and a hit in the NDIS (National DNA Index System) [6,8]. This equates to one new sexual assault offense every 51.41 days a sexual predator is at large (365 days per year/7.1 sexual assaults per year), which could be prevented with the application of forensic analysis and databank technology currently in place. This means that for each recidivist offender, a new preventable victim arises every 51.41 days a forensic case sits in an awaiting analysis to commence backlog.

There was a time where previously unanalyzed cases awaited a technology to permit their resolution. Now that the technology has arrived, there is a new challenge to optimize response time to take advantage of the value of the system now put in place [3,4]. The existence of a backlog of cases awaiting analysis provides additional opportunity for an unapprehended perpetrator to continue committing offenses. Each additional day at large represents preventable crime, and hence savings opportunity.

Previous studies estimate the average output of a forensic DNA analysis at 96 cases annually [7,13]. Project Foresight includes metrics provided by 168 forensic laboratories [14]. The median output for a DNA analyst in Project Foresight participating laboratories is 102 cases [14]. It is noteworthy that with economies of scale applied to forensic casework, optimal output could be significantly higher. However, as jurisdictions are fixed and cases are frequently not mobile between forensic laboratories, achieving the optimum output may be inhibited.

With approximately 220 productive work-days available annually, this equates to approximately 0.4636 cases output per analyst per day. There are 203 CODIS laboratories in the US [15]. In the last survey of publicly funded forensic crime laboratories conducted in 2014, the median number of employees was 20 [16]. Assuming a small to medium sized forensic laboratory had 10 DNA analysts working on sexual assault cases full time, their combined output would be approximately 4.63 cases per day. With this assumption that the average forensic DNA laboratory has 10 analysts, there would be approximately 2030 forensic DNA analysts (203 laboratories X 10 analysts) in CODIS laboratories the United States at the last industry-wide survey in 2014.

In a sexual assault case backlog reduction initiative between 2015 and 2018, the Palm Beach County Sheriff’s Office obtained CODIS eligible profiles in 44% of sexual assault cases and found a 36% hit rate for those eligible CODIS profiles [17]. This computes to a 15.8% hit rate on the total number of sexual assault cases analyzed. In a sexual assault cold case initiative conducted by Acadiana Criminalistics Laboratory (ACL) in Louisiana, 47% of cases analyzed produced a CODIS eligible profile and realized a 57.54% hit rate [5]. In a Detroit sexual assault kit backlog reduction initiative, 1595 cases were tested, which resulted in 785 eligible CODIS profiles and 455 hits [9]. This equates to a 57.96% hit rate on CODIS profiles entered and a 28.53% hit rate per sexual assault kit tested. The ACL initiative utilized preserved sample cuttings taken from the analysis of sexual assault cases, while the Palm Beach and Detroit projects utilized sexual assault cases themselves. Hence, the cases examined in each of these projects are relatively comparable, and a reasonable approximation of expectations for successful outcomes. The Project Resolution Louisiana Cold Case project generated a hit in 27.11% of the total number of sexual assault cases analyzed (164 hits in 605 cases) [5].

Using these three forensic laboratories and their cold case analysis outcomes as benchmarks, they produce an average 24.13% hit rate for sexual assault cases analyzed (see Table 1). Using an estimated output of 0.4636 cases per day means that the average analyst day would produce 0.1119 hits per day (0.4636 cases per day X 0.2413 average hit rate), or 24.6 hits per year (0.1119 hits per day X 220 workdays/year) at their published level of output for a single DNA analyst (see Table 2).

**Table 1:** Sexual assault cold case DNA recovery and hit rates.

Forensic Laboratory	Cases Completed	CODIS Profiles	Hits	Recovery Rate	Hit Rate per CODIS Entry	Hit Rate per Sexual Assault Case
Acadiana Criminalistics Lab Laboratory [5]	605	285	164	47.11%	57.54%	27.11%
Palm Beach Sheriff's Office Crime Laboratory [17]	1558	686	261	44.03%	38.05%	16.75%
Detroit; Michigan State Police Forensic Science Division (9)	1595	785	455	49.22%	57.96%	28.53%
Mean				46.78%	51.18%	24.13%

**Table 2:** U.S. per DNA analyst CODIS hit impact of the awaiting analysis backlog.

Average DNA Hit Percentage	24.13
Average Case Output per Analyst Annually (14)	102
CODIS Hits per Analyst per Year	24.6
Average Case Output per Analyst Daily	0.463
CODIS Sexual Assault Hits per Day per Analyst	0.1119
CODIS Sexual Assault Hits in 30-day Awaiting Analysis Backlog	3.36

Two thousand and thirty DNA analysts would produce approximately 227 hits daily (0.1119 hits per day X 2030 analysts), or 49,964 hits annually (102 cases per year per analyst X 2030 analysts X 0.2413 hit rate). As of April 2021, CODIS has produced over 562,412 hits assisting in more than 549,516 investigations [18]. CODIS became fully operational in 1998, starting initially as a pilot program, with hits increasing as the size of the database increases [19]. The accelerating number of CODIS hits as the database grows supports the estimate of 49,964 hits on sexual assault cases nationally. With each single day of national output yielding an estimated 227 hits on sexual assault cases, it stands to reason that reducing the time a case sits awaiting analysis by one day would produce a corresponding 227 additional CODIS hits. If each forensic laboratory had an average of 30 days of backlog of cases awaiting analysis to commence, that is an additional 6810 cases (227 hits per day X 30 days) that could be provided investigative leads. This additional time a case awaits analysis being started represents opportunity for the commission of additional crimes, which could be prevented if the suspect was no longer at large.

Two different models will be applied to provide a cost of crime estimate arising from the resolution of a sexual assault case [6,7]. The models differ in the estimated cost of crime and the estimated number of potential sexual assault cases that could be prevented if a case is solved in a timely manner. These two models will be termed the conservative model and aggressive model based on the level of cost and recidivism provided in each. The conservative model of the cost of crime applied a dollar figure of $111,238 per sexual assault [7], while the more aggressive model was $435,419 [6]. The conservative model applies a recidivism factor of 7 cases prevented for each crime matched by CODIS [7] while the aggressive model applied a cases prevented recidivism factor of 26.22 [6]. The average expense for analyzing a sexual assault case has been estimated at $1641 [6]. Additional cost estimates averaged over multiple forensic laboratories are provided in the Annual Project Foresight Report [20]. The median cost cited for a DNA case is $1411 and for a Serology/Biology case is $1035. The range of costs of DNA varied over the past 5 years between a high of $1996 in 2016–2017 and a low of $1299 the following year. In 2019–2020 the range of DNA analysis cost was $1102 for the 25th percentile to $2185 at the 75th percentile. As sexual assault kits represent an aspect of both Serology/Biology and DNA and are a specific subset of Biology Section casework, the more specific number of $1641 will be applied, noting there is a significant range for costs between forensic laboratories, including variations across the last 5 years of Project Foresight published data and this point estimate falls well in line with available data [20]. With an average output factor of 102 cases per year [14] based on a 220-workday year, the daily expense of DNA analysis computes to roughly $761 per day ($1641 case expense X (102 cases per analyst per day/220 days)) (see Table 3).

Using the conservative cost of crime model [7], each day of backlog reduction could save $12,447 ($111,238 cost per sexual assault X 0.1119 hits/day) for each DNA analyst, if one sexual assault was prevented per CODIS hit. Applying the recidivism factor of 7, the return on investment rises to $87,129 per day ($12,447 saving X 7 cases prevented per CODIS hit). With an estimated expense of $761 per analyst day, this equates to savings of $113.50 per dollar spent (($87,133 saving-$761 daily expense)/$761 daily expense) (see Table 4) or a 11,350% return on investment per day of reduced turn-around time.

**Table 3:** U.S. Nationwide CODIS hit impact of the awaiting analysis backlog.

Number of Forensic DNA Laboratories	203
Average Analysts per Laboratory	10
Total Number of DNA Analysts in USA	2030
Average DNA Hit Percentage	24.13
Average Case Output per Analyst Annually	102
CODIS Sexual Assault Hits per Year Nationally	49,964
CODIS Sexual Assault Hits in 30-day Awaiting Analysis Backlog	6821

**Table 4:** Cost of crime savings per DNA analyst day of reduced awaiting analysis backlog.

	Cost of Sexual Assault	CODIS Hits per day	Analysis Cost per day	Recidivism Factor	Return on Investment	Total saving per analyst per day	Total saving nationally per day turn-around time reduction	National saving for 30-day turn-around time reduction
Conservative Model	$111,238	0.1119	$761	7	$113.50	$87,133	$176,879,990	$5,306,399,700
Aggressive Model	$435,419	0.1119	$761	26.22	$1677.75	$1,277,527	$2,593,379,810	$77,801,394,300

By contrast, applying the more aggressive model [6] each DNA analyst workday of backlog reduction would save $48,723 ($435,419 cost per sexual assault X 0.1119 hits/day) if one sexual assault could be prevented. Applying the recidivist factor associated with the aggressive model of 26.22 preventable sexual assaults per CODIS hit and the potential savings per day of reduced turn around time increases to $1,277,517 ($48,723 saving X 26.22 cases prevented per CODIS hit). With an analyst cost per day estimated at $761, the return on investment is $1677.75 for every dollar spent (($1,277,517 saving-$761 daily expense)/$761 daily expense) (see Table 4), or 167,775% return on investment per day of reduced turn-around time.

The cost-saving per analyst day was estimated at between $87,133 (conservative model) and $1,277,527 (aggressive model) represent significant savings. The return on investment (ROI) is estimated at between $113.50 and $1677.75 for every dollar spent (see Table 4), or between a 11,350% and a 167,775% return on investment per day of reduced turn-around time. This cost-benefit demonstrates the large cost of cases sitting awaiting analysis to begin. This cost could be prevented by elimination of the awaiting analysis backlog. With these estimates for a single analyst, the savings taken on a nationwide basis are much larger. With an estimated 2030 DNA analysts nationwide, each day of reduced awaiting analysis backlog could save between $176.8 Million ($87,133 cost saving X 2030 analysts nationally) and $2.59 Billion ($1,277,527 × 2030 analysts nationally). If U.S. forensic laboratories nationwide had an average of a 30-day awaiting analysis backlog, starting cases immediately upon submission would save between $5.31 Billion ($176,879,433 saving X 30 days) and $77.8 Billion ($2,593,380,183 saving X 30 days).

As noted in a previous publication, the 30-day turn around time is steeped in forensic history [21]. When a large backlog contained the assailant in a string of serial sexual assaults in Canada, two preventable homicides resulted while the perpetrator was at large. The resulting inquiry provided a recommendation of a 30-day turn-around time target to prevent additional crimes resulting from large backlogs impeding investigations. Noting this case occurred in the 1990’s, technology has improved markedly, enabling much quicker forensic analytic response times. Many forensic laboratories still adhere to 30 days to begin counting their backlogs to account for cases in analysis, and it has been noted that this backlog has begun to increase [22]. Therefore, 30 days is utilized as an illustration of the potential savings for full implementation of eliminating the awaiting analysis backlog nationally, acheived by starting analysis on cases as they enter the crime laboratory.

The above calculations provide a snapshot in time to illustrate the tremendous potential savings of reducing the awaiting analysis backlog, while in reality the true backlog situation is much more dynamic. While a backlog of crimes represents forensic cases yet to be processed, the databank includes known individuals which are necessary for comparison to the unknown forensic profiles. Each side of the coin, forensic profiles, and databank samples are necessary to optimize success. Recent studies indicate a potentially larger impact than provided here given a larger variety of crime types and increase in database size, reducing recidivism by 42% [23]. Registrants experienced improved education, employment, and family environment outcomes. Solving past crimes and inclusion in databases prevents crimes through apprehension, deterrence, and correction.

## 3 Case of the Two Marks

Much has been made regarding the power of forensic comparison and investigative leads to include suspects. The ROI dollar value provides a universal measure to demonstrate the crime preventing potential of timely forensic analysis. For each suspect who is included, particularly if a database was utilized, many individuals who are not a match were eliminated as potential suspects. The justice system must be directed by science and data, to prevent false convictions, miscarriages of justice, false charges, misplaced suspicions, and the costs they convey. Suspicion of crime carries a heavy weight, one which should be lifted as soon as is feasible given existing forensic technology. The duty to eliminate and exonerate innocent individuals from suspicion of crime cannot be overstated. The Innocence Project uses forensic tools to demonstrate innocence, especially when DNA is located in a probative crime scene location [24]. Such case successes must be celebrated. However, the best exoneration is one that did not occur, as a suspect was eliminated from suspicion early in their case, rather than post-conviction.

One particularly poignant case demonstrates the value of objective forensic data in supporting both eliminations and inclusions. The case of the “Two Marks” involves two males both with the given name Mark. In a sexual assault case occurring in 1995 in Upstate New York, a woman met a man named Mark at a bar. Her acquaintance Mark invited her outside of the bar to smoke marijuana. She subsequently left the bar with him. Once outside and away from public view, during an ensuing struggle, Mark bound her with a belt and sexually assaulted her. Once the ordeal ended, she escaped and reported the crime to police. Police investigators accompanied the survivor to a hospital, where an examination was performed, and a sexual assault kit was collected. Based on the survivor’s description and that of other bar patrons, a suspect with the first name of Mark was identified, arrested, and charged with the offense. It was noted that the suspect Mark had scratches on his forearms, which fit the story of the struggle which was put up by the sexual assault survivor.

As part of a thorough investigation, police investigators submitted the sexual assault kit to the New York State Police Forensic Investigation Center in Albany, New York. A forensic analyst was assigned the case, examined the sexual assault kit, identified spermatozoa, and developed a DNA profile from the forensic sample. A comparison between the known profile and the forensic profile conclusively eliminated the first suspected individual named Mark. With additional police investigation a second suspect, also with the given name Mark, was developed. A biological sample was taken from the second Mark and submitted to the forensic laboratory. A DNA profile was developed and compared to the forensic DNA profile from the sexual assault kit. The DNA profile from Mark #2 matched the forensic sample. When presented with the evidence, the second Mark plead guilty to the crime. The resemblance between the two different individual suspects named Mark was remarkable.

While much focus is placed on the crime solving capability of forensic tools such as DNA, the ability to eliminate wrongfully suspected individuals cannot be underestimated. In the days prior to the availability of such forensic tools, the chances for the first Mark to effectively proclaim his innocence would have been severely compromised in the absence of such conclusive demonstrations of his non-involvement in the crime. One might assume that the first Mark could quite easily have been wrongfully convicted of the crime, particularly based on eyewitness testimony of the survivor, corroborated by the other bar patrons. While difficult to place a dollar value on an elimination, multi-million-dollar settlements on wrongful convictions appear to be doing just that [24].

## 4 Discussion

The ROI estimations provided herein demonstrate the cost savings of reducing awaiting analysis backlog and improving turnaround time are very large, particularly when taken on a U.S. nationwide scale. Elimination of the bottleneck of DNA cases with perpetrator DNA present sitting awaiting analysis while the perpetrator remains at large represents a rare opportunity to take advantage of existing forensic technology. Survivors of crime are owed more than a good faith effort toward resolution of their case. They should be assured that learning from past experiences have put a system in place that would prevent their ordeal from reoccurring needlessly on new victims. The CODIS system recently on April 21, 2021 passed a landmark of 20 Million entries and over 545,000 investigations aided through DNA hits [15]. The investment in building the technology and infrastructure of NDIS/CODIS has taken the better part of three decades. The use of this established tool must be optimized to realize the savings to the victims of crime as demonstrated by these multi-billion-dollar savings potentials by preventing crime.

There are more dimensions to forensically driven investigation than simply identifying an individual of interest. Inherent in that identification, is that many other individuals are eliminated. A multitude of individuals included in CODIS have DNA profiles different from the crime scene sample, and therefore can be effectively eliminated as suspects, particularly when the piece of evidence is incriminating. This feature provides justification for using no-suspect sexual assaults as a model for the business case examples demonstrated herein. Where an item of evidence is very probative, as in the individual who deposited the DNA is very likely the individual who committed the crime, forensic science is particularly valuable as an instrument of elimination as it is inclusion. Not all criminal cases have the advantage of such significantly probative evidence. Additional investigation beyond forensic results should be conducted in all criminal cases, particularly regarding the location of the individual with respect to the timing of the crime to demonstrate access and opportunity.

Both sides of the forensic science equation are compelling. Recidivist perpetrators at large exact a heavy toll on victims with additional preventable crimes. Existing technology that has shown its worth, both in including and eliminating suspects. A case of elimination can be argued to be worth far more than an inclusion in the American system, as the Blackstone principle instructs that in distributing errors in criminal punishment, the U.S. justice system should strive to minimize false convictions, even at the expense of creating more false acquittals and more errors overall [25].

Computing a dollar value to demonstrate value provides a universal measure that enables comparison among competing priorities for rare resources. While the model utilizes sexual assault cases to demonstrate cost, every forensic discipline provides similar value by including or eliminating individuals from suspicion. Protecting public safety and individual rights rank among the highest concern taxpayers have for expenditures of their tax dollars. Issues raised by both Black Lives Matter and victim advocates for survivors of sexual assault are supported through providing science-based evidence earlier in investigations. There really is no downside in using existing forensic technology more quickly, other than the increased resources to deliver necessary analytical capacity.

What is not fully captured in reducing the damage and cost created by crime to a dollar amount is the human toll that it exacts. The investigative system’s goal is justice, not strictly solving a crime, but rather swiftly eliminating suspects, directing investigations towards the correct suspect, and minimizing danger to society. That danger includes both preventable victimization from the crime itself as well as its investigation. Furthermore, the crime survivor is subjected to an increased burden without corroborating evidence, to recollect a most stressful event in their lives repeatedly through investigation and court testimony, with the added pressure of not wrongfully accusing an innocent individual. Such was the situation in a sexual assault case involving a wrongfully accused individual Ronald Cotton who was incorrectly identified by the crime survivor [26].

As a society, we must find a balance of rights, respecting each individual’s autonomy; their right to live their lives free of both crime and wrongful accusation. Our laws and the mechanisms for protecting and enforcing them must support our ideals. The George Floyd case and the COVID-19 pandemic have both shown us that facts and data, when we all see the same thing, guides our way to the best solution. Better information and solid verifiable facts lead to better decision making and a more just and equitable society. Victims of crime have a right to have their case analyzed quickly and transparently. So too do those who would be wrongfully accused if not for timely analytical results. Innocent Mark must be saved as early in the process as possible.

If one is convinced that public safety and individual rights and freedoms are best served by providing the quickest response time available for forensic analysis, three questions arise. These questions are first what is the responsibility of existing forensic laboratories? Secondly, what is the responsibility to those who oversee and resource forensic laboratories? The third question is what should be done to provide the most beneficial forensic analytical response?

In response to the first question regarding the role of forensic laboratories, as the stewards of government resources afforded for forensic services, forensic laboratories have responsibility to make the best use the resources entrusted to them. They must obtain maximum efficiency while maintaining quality. This can be achieved in part by reducing examinations to only those most probative, thereby only applying analysis to items where results will guide investigations [21]. The latest techniques should be applied to maximize the value of evidence for both exclusionary and inclusionary potential. Workflow must be optimized, using Lean six sigma principles to reduce waste and redundancy, while maximizing quality and output. Updated LIMS (Laboratory Information Management Systems) should be used to facilitate and document workflow and examinations, providing electronic reporting to reduce turnaround time. Once processes are optimized, forensic laboratories have a duty to advocate for additional resources to eliminate the backlog of cases awaiting analysis to commence, as it has been demonstrated that timely analysis serves survivors of crime, public safety through the reduction of preventable crime, and the wrongfully accused.

A response to the second question requires a look at the current state of US forensic laboratories. The vast majority of the 409 publicly funded forensic laboratories in the United States operate independently [16]. Most forensic laboratories in the U.S. provide services within state and local jurisdictions. The median number of employees in U.S. forensic laboratories is 20 [16]. For many forensic laboratories, their small size makes keeping their expertise and technology current a significant challenge.

Significant federal funding has been provided through grants including the DNA Capacity Enhancement for Backlog Reduction Program, which has been instrumental in building U.S. forensic technology and capacity [27]. However, there is no centralized government structure to develop, implement and administer forensic laboratory analyses. There exists a tremendous opportunity to apply national economies of scale to training, validation, acquisition and implementation of new equipment, technology, and LIMS (Laboratory Information Management Systems). These and many other efforts duplicated in forensic laboratories could be made more efficient by applying common models and shared best practices. Redundancies in programs do more than duplicate efforts, they miss the opportunity to benefit from shared experience to further hone common methods and programs [29].

Many forensic scientists provide volunteer expertise through forensic associations and standards developing organizations and initiatives. While there are small dedicated corps of full-time employees facilitating grants and the development of standards, and supporting forensic DNA technologies with scientific expertise, there is no central guidance coordinating and conducting the business of delivering forensic science service for the entire U.S. forensic enterprise. While forensic scientist volunteers add great value in ensuring standards are developed that are fit for purpose and there is opportunity for sharing expertise, there exists a much larger opportunity to identify and direct resources to more effectively support forensic science quality, improved technology, and performance vis-à-vis the reduction of turnaround time.

The National Institute of Science and Technology (NIST) houses the Applied Genetics Group, whose mission is to “to focus on developing standards and technology to aid human, plant, and animal identification and to benefit agricultural, law enforcement, and clinical applications using genetic information [28].” Assessing and validating new technologies is a costly venture, currently being duplicated across the 409 forensic laboratories in the U.S. The core competency of forensic laboratories is conducting forensic analysis, rather than taking time away from casework analysis to assess, acquire, validate, and implement new technology. The value of taking time away from casework to conduct validation studies has been demonstrated to be very costly [29] and would perhaps be better placed within a centralized unit which could coordinate with forensic laboratories to avoid duplication while developing best practices and validated centralized test methods. Forensic laboratories are free to establish their own programs. However, adopting existing methods validated and published by a recognized and independent scientific entity would permit uninterrupted casework output and focus on backlog reduction to continue. This independent entity could be the NIST Applied Genetics Group or similar entity for other forensic disciplines. Another benefit would be the opportunity to centralize coordinated training, including the development of online training materials and the integration of methods meeting OSAC approved standards [30].

A centralized office to provide support, guidance, and coordinate teams of experts to visit, assess, validate, implement and train has immense potential to dramatically improve forensic service by augmenting existing programs. The COVID-19 pandemic has sped the development of online tools and capability to share resources, conduct on-line training and meetings. There is a need for direct contact with laboratories and their staff, including direct observation, assessment, guidance, training, and technology transfer. Peer scientist mentors (outside in) and visiting scientists (inside out) leverage and develop professional relationships and apply direct observation and hands-on experience to facilitate improvements.

Forensic laboratories and their experts possess tremendous scientific expertise. However, their sharing of talent should not be left to chance, but rather organized and focused on implementation by assisting peers directly in a coordinated and appropriately resourced effort. Rather than the current ad hoc approach, there is an opportunity for a national solution to design and apply resources to fit the problem in a customized and focused manner. Therefore, the response to the second question regarding the responsibility to those who oversee and resource forensic laboratories is to provide coordinated and educated guidance through an office of forensic science.

Finally, in response to the third question, what should be done? With a centralized office in place to coordinate efforts, a gap analysis can be conducted to determine the delta between the current state of forensic service and the desired state. The forensic community has already undertaken significant efforts in this regard which can inform next steps. A forensic needs assessment was completed in 2019, which details forensic needs, challenges, and promising practices [31]. Published testimony before the Presidential Commission on Law Enforcement and the Administration of Justice on April 14, 2020 by Matthew Gamette, Director of the Laboratory System for the Idaho State Police Forensic Services and the Chair of the Consortium of Forensic Science Organizations includes a perspective on many opportunities for forensic improvement on domestic abuse and sexual assault investigations [32]. Dr. Paul Speaker of West Virginia University is the principal investigator responsible for Project Foresight [33], which includes the LabRAT form for generating benchmarking reports. The project provides detailed estimates for forensic resources and optimization, which provide a wide basis for data to generate resource need estimates. RTI International hosts the Forensic Science Technology Center of Excellence, which provides a workforce calculator tool to estimate the full slate of required personnel resources to address estimated caseloads [34]. The tool includes provision for staffing of necessary support and administrative positions, which are required to enable forensic analysts to focus specifically on casework analyses. While these proposals and tools are not exhaustive, they provide a roadmap with data to guide a more comprehensive coordinated approach than the current more laisse faire approach to forensic service provision in the United States.

Forensic technology is now here. We now need to design the appropriate fit of supply to meet the demand. Survivors and the wrongfully accused deserve no less.

## 5 Conclusion

The value of forensic analytical results to solve crime earlier has been demonstrated by a large return on investment. A conservative model calculates savings of $113.50 per dollar spent versus a more aggressive model with savings of $1677.75 per dollar spent for each day earlier forensic analytical results can be delivered. If U.S. forensic laboratories nationwide had an average of a 30-day awaiting analysis backlog, starting cases immediately upon submission would save between $5.31 Billion and $77.8 Billion. These results have significant implications for forensic laboratories and policy makers. Forensic laboratories must use every method within their disposal to shorten the timeline between case submission and the generation of reports, including streamlining laboratory operations, focusing analyses on the most probative cases and items and utilizing LIMS (Laboratory Information Management Systems) to streamline reporting electronically. Ideally, these systems should be in various stages of implementation as forensic laboratories strive to provide the best possible service for the resources they have been entrusted. Therefore, a broader national coordinated application of resources is supported to deliver results in the timeliest fashion to maximize the value of existing forensic technology. The quickest a case can be completed is if analysis is started when the case arrives at the lab, versus sitting awaiting analysis to start due to a backlog produced by a shortage of resources. The good news is the technology is available to solve and exonerate. The bad news is, as is true with most technology, you get what you pay for. Shortcuts should not be taken on quality nor service. The mission of the forensic laboratory is to maximize the value of evidence, which is accomplished by delivering results as quickly as technology allows. Resources targeted with coordinated programs leveraging existing expertise appears to be a part of a well-crafted solution. Preventing crime and eliminating the innocent serves all of us well.

## Declaration of competing interests

The authors declare that they have no known competing financial interests or personal relationships that could have appeared to influence the work reported in this paper.
